# The effect of *Vaccinium uliginosum* extract on tablet computer-induced asthenopia: randomized placebo-controlled study

**DOI:** 10.1186/s12906-016-1283-x

**Published:** 2016-08-18

**Authors:** Choul Yong Park, Namyi Gu, Chi-Yeon Lim, Jong-Hyun Oh, Minwook Chang, Martha Kim, Moo-Yong Rhee

**Affiliations:** 1 Department of Ophthalmology, Dongguk University, Ilsan Hospital, Goyang, Gyeonggido South Korea; 2 Department of Clinical Pharmacology and Therapeutics, Dongguk University, Ilsan Hospital, Goyang, Gyeonggido South Korea; 3 Department of Biostatistics, Dongguk University Ilsan Hospital, Goyang, Gyeonggido South Korea; 4 Department of Cardiology, Dongguk University, Ilsan Hospital, Goyang, Gyeonggido South Korea; 5 Department of Ophthalmology, Dongguk University, Ilsan Hospital, 814, Siksadong, Ilsan-dong-gu, Goyang, Kyunggido 410-773 South Korea

**Keywords:** Asthenopia, Antioxidant, Computer vision syndrome, Billberry, *Vaccinium*

## Abstract

**Background:**

To investigate the alleviation effect of *Vaccinium uliginosum* extract (DA9301) on tablet computer-induced asthenopia.

**Methods:**

This was a randomized, placebo-controlled, double-blind and parallel study (Trial registration number: 2013–95). A total 60 volunteers were randomized into DA9301 (*n* = 30) and control (*n* = 30) groups. The DA9301 group received DA9301 oral pill (1000 mg/day) for 4 weeks and the control group received placebo. Asthenopia was evaluated by administering a questionnaire containing 10 questions (responses were scored on a scales of 0–6; total score: 60) regarding ocular symptoms before (baseline) and 4 weeks after receiving pills (DA9301 or placebo). The participants completed the questionnaire before and after tablet computer (iPad Air, Apple Inc.) watching at each visit. The change in total asthenopia score (TAS) was calculated and compared between the groups

**Results:**

TAS increased significantly after tablet computer watching at baseline in DA9301 group. (from 20.35 to 23.88; *p* = 0.031) However, after receiving DA9301 for 4 weeks, TAS remained stable after tablet computer watching. In the control group, TAS changes induced by tablet computer watching were not significant both at baseline and at 4 weeks after receiving placebo. Further analysis revealed the scores for “tired eyes” (*p* = 0.001), “sore/aching eyes” (*p* = 0.038), “irritated eyes” (*p* = 0.010), “watery eyes” (*p* = 0.005), “dry eyes” (*p* = 0.003), “eye strain” (*p* = 0.006), “blurred vision” (*p* = 0.034), and “visual discomfort” (*p* = 0.018) significantly improved in the DA9301 group.

**Conclusions:**

We found that oral intake of DA9301 (1000 mg/day for 4 weeks) was effective in alleviating asthenopia symptoms induced by tablet computer watching.

**Trial registration:**

The study is registered at www.clinicaltrials.gov (registration number: NCT02641470, date of registration December 30, 2015).

## Background

Asthenopia or eye strain is defined as nonspecific symptoms of an eye when it is tired from intensive work [1]. The symptoms of asthenopia are diverse and include fatigue, pain, blurred vision and headache.

Several extrinsic and intrinsic factors have been known to cause asthenopia [1, 2]. The common causes of asthenopia are, but not limited to, reading, computer work, dry eye, presbyopia and uncorrected refractive errors. In addition, asthenopia is one of the important subjective symptoms for the diagnosis of dry eye syndrome [3]. Asthenopia induced by computer screen watching is known as computer vision syndrome (CVS) [4, 5]. Symptoms that constitute CVS include ocular, visual and musculoskeletal discomfort. Wide-spread use of mobile devices including smart phones and tablet computers has raised great concerns about the potential ocular side effects caused by long-term use of these devices [6–8]. Continuous watching of computer screen is closely related to decreased blink rate and increased accommodation and convergence activities, which can lead to eye dryness and asthenopia [9, 10]. In addition, recently available mobile devices adopt light emitting diode (LED) display terminals. Although LED can provide higher resolution images, a short wavelength of light, especially blue range light, emitted from LED can damage retinal cells [11, 12]. Therefore, these all changes occurring during computer monitor watching can manifest as complex symptoms of CVS.

*Vaccinium uliginosum* is a flowering plant native to low-temperature regions of the Northern Hemisphere. It is found in the Alps, the Pyrenees, the Caucasus in Europe as well as in Mongolia, China, Japan and Korea [13]. The extract of *Vaccinium uliginosum* fruit contains abundant antioxidant compounds such as anthocyanins and flavonoids [14, 15]. Antioxidants supplementation has been known as effective in alleviating dry eye symptoms [16–18]. In addition, the extract of *Vaccinium uliginosum* demonstrated protective effect of retinal cells in light-induced retinal damage [19]. Considering its antioxidative constituents and previous evidences of retinal protection, we hypothesize that the extract of *Vaccinium uliginosum* will be useful in treating various ocular pathologies including computer vision syndrome.

We performed a randomized, placebo-controlled study in healthy subjects and investigated the protective effect of *Vaccinium uliginosum* extract (DA-9301) on tablet computer-induced asthenopia.

## Methods

### Study design and population

This is a randomized, placebo-controlled, double-blind, parallel design study. The study was conducted at Clinical Research Center located in Ilsan Hospital, Dongguk University, Goyang, South Korea and followed the tenets of the Declaration of Helsinki and was approved by the institutional review board (IRB) of Ilsan Hospital, Dongguk University (IRB approval number: 2013–95). The study is registered at www.clinicaltrials.gov (registration number: NCT 02641470). Written informed consents were obtained from participants before their enrollment. The screening for the first volunteer was started on February, 2014 and the whole study was ended on May, 2014.

**Fig. 1:**
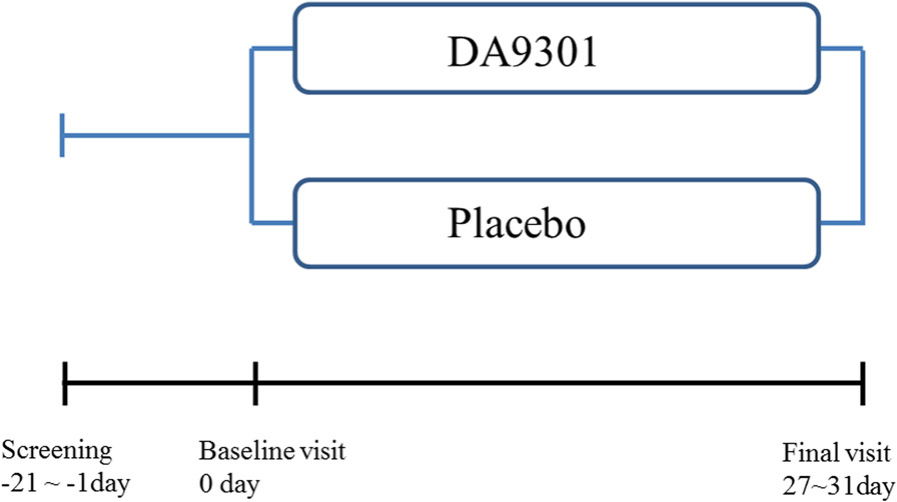
Schematic design of the study

Sixty two volunteers were screened and 60 eligible volunteers were randomized. The inclusion criteria were healthy subject aged 20–65 years with more than 2 h of daily use of a smart phone or computer including a tablet and television. Exclusion criteria are listed in Table 1. After passing the screening test (two subjects were excluded as screening failure), 60 subjects were randomized to study (DA9301) and control groups in double blinded manner at a ratio of 1:1. The randomization codes were generated using a block algorithm by a statistician and the seed number for the algorithm was the date that the codes were created. The study group received DA9301 pills (1000 mg/day) for 4 weeks and the control group received placebo (same shape, size, and color as those of the DA9301 pill). (Figure 1) Now the study group is referred as DA9301 throughout the manuscript. Compliance was measured both by reviewing the drug intake diary and by counting remnant pills at the final visit. If the compliance failed to reach 80 %, the participants were excluded. In addition, any participants who took forbidden systemic medications such as antihistamines, anticholinergics, thyroid hormones, anti-thyroid drugs and *Ginko biloba* extracts were excluded. These medications were forbidden because they may affect ocular surface condition during the study period [20–23].

**Table 1:** Exclusion criteria

-Ocular disease in either eye a. Ocular surface disease Corneal fluorescein staining grade ≥3 Tear film break up time <5 s Schirmer I (without anesthetic) test <5 mm b. Best corrected visual acuity < 20/30 c. Intraocular pressure > 21 mmHg d. Optical coherence tomography proven retinal nerve fiber defect e. Significant cataract (lens opacities classification system III: ≥NO3/NC3, ≥C3 or ≥ P3) f. Significant entropion or ectropion g. Significant tear drainage problem proved with fluorescein dye dilution test -Soft or Hard contact lens use 3 or more days a week -History of oral intake of health supplement designed to improve asthenopia within 4 weeks before participating this study -Regular use of any lubricant eye drops -Pregnant woman -Systemic disease a. Uncontrolled hypertension (systolic/diastolic blood pressure > 140/90 mmHg) b. Uncontrolled diabetes mellitus (fasting blood glucose level > 180 mg/dL) c. Rheumatoid arthritis d. Malignant disease e. Active hepatitis (type B and C) f. Acute or chronic infectious disease g. Renal disease

### Preparation of Vaccinium ulginosum extract (DA9301)

The fruits of *Vaccinium uliginosum* was washed and extracted with water at 60 ~ 70 °C, and the extracts were subsequently went through diatomaceous earth filtration. The filtered extracts were concentrated and sterilized at 50 °C, 60 ~ 65brix. Diluent (Maltodextrin) was added to the extracts and sterilized again at 80 ~ 95 °C for 10 min for two times. And it was spray-dried. The marker compound of Vaccinium uliginosum is total polyphenols. Its content is approximately 9.1 mg/g, acceptable within a range (80 % to 120 %).

### Preparation of DA9301 and placebo pills

The DA9301 pill was prepared by mixing DA9301 (500 mg), lactose (56.63 mg) and crystal cellulose. The placebo pill only contained lactose (556.63 mg). The shape, size, and color of both the DA9301 and placebo pills were the same.

### Ophthalmic screening test

Ophthalmic screening for participants included slit lamp microscopy, non-contact intraocular pressure measurement, fundus photography, corrected distance visual acuity, Schirmer’s test, and tear-film break-up time.

### Tablet computer screen stimulus

Participants in the DA9301 and control groups were asked to watch a table computer screen (iPad Air, Apple Inc., Cupertino, CA) for 1 h at a viewing distance of 40 cm at baseline and 4-week visits. Participants watched a movie or played a computer game on the tablet computer. Those with presbyopia were allowed to use reading glasses for their comfort. For exact comparisons, subjects who watched a movie or played a game at baseline visit were asked to repeat the task at 4 week visit. Tablet computer screen stimulation was performed in the same room during all the study period to consistently maintain the humidity, temperature and illumination; the humidity and temperature were checked on every study day.

### Outcome measures

Asthenopia was evaluated by using a modified questionnaire proposed by Ames et al. (Table 2) [24]. These questionnaires contained 10 questions related to asthenopia that needed to be responded to using a scale of 0–6, with 0 defined as none and 6 as most severe. Most severe asthenopia corresponded to a score of 60. All subjects completed the questionnaire before and immediately after tablet computer use. The severity of asthenopia may change depending on daily body condition including diurnal variation and environmental factors such as illumination, humidity and room temperature [2, 25]. Therefore, to compare the effect of tablet computer watching, the asthenopia scores before and immediately after tablet computer watching (induced asthenopia) were calculated and score change was compared between the DA9301 and control groups. For objective measurement of ocular dryness and visual deterioration, tear film break-up time and total high order wavefront aberration (Wavescan, VISX/Advanced Medical Optics, Santa Clara, CA) were measured in the right eyes before and after tablet computer watching. The measurements were made triplicate and the mean values were used for the analysis.

**Table 2:** Asthenopia questionnaire form modified from the original version proposed by Ames et al.

Symptom	None	Slight		Moderate		Severe	
Tired eyes	0	1	2	3	4	5	6
Sore/aching eyes	0	1	2	3	4	5	6
Irritated eyes	0	1	2	3	4	5	6
Watery eyes	0	1	2	3	4	5	6
Dry eye	0	1	2	3	4	5	6
Eye strain	0	1	2	3	4	5	6
Hot/burning eyes	0	1	2	3	4	5	6
Blurred vision	0	1	2	3	4	5	6
Difficult focusing	0	1	2	3	4	5	6
Visual discomfort	0	1	2	3	4	5	6

### Dietary antioxidant measurement

Participants presented the last 3 days diet before the 4 week visit. Nutritional components within diet were analyzed by using computer aided nutritional analysis program (CAN) pro v4.0 devised by Korean Nutrition Society. Vitamins C and E, beta-carotene and selenium were selected as potential antioxidant nutrients and their uptake was compared between DA 9301 and placebo groups.

### Statistical analysis

A total of 50 patients (25 patients in each group) were needed to achieve 80 % power and to detect a difference of 0.72 in the total score of asthenopia questionnaire. To allow for 15 % drop-out rate, we randomly assigned 30 participants to each group (study vs. control). Statistical analysis was performed using SAS® (Version 9.3, SAS Institute, Cary, NC). An assessment of normality of data was assessed by using Shapiro-Wilk test. The comparisons between the study groups were performed with the use of Student’s *t*-test (or Wilcoxon’s rank sum test) and paired *t*-test (or Wilcoxon’s signed rank test) for continuous data and the chi-square test or Fisher’s exact test for categorical data.

## Results

**Fig. 2:**
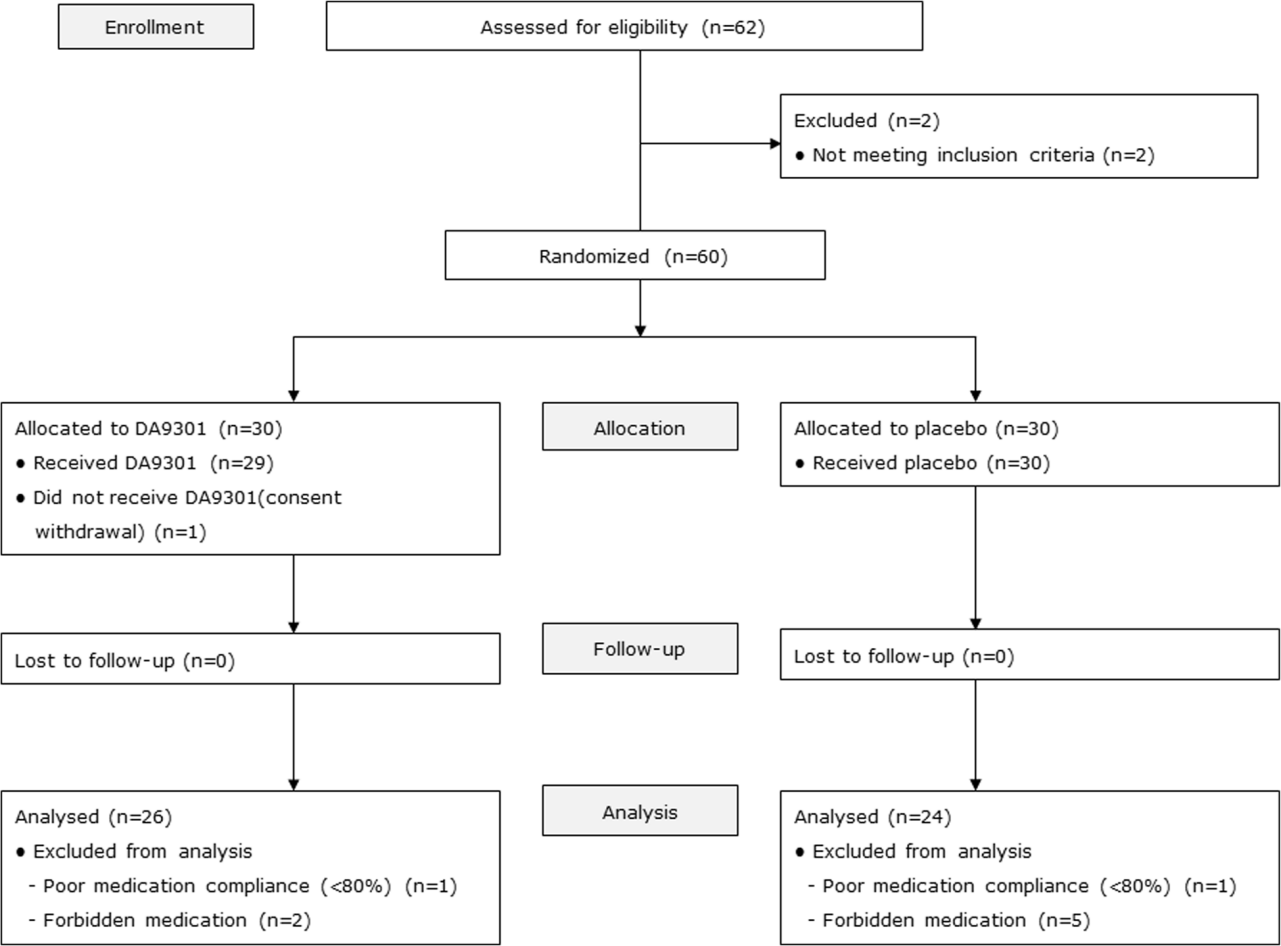
Flowchart (CONSORT) of the participants throughout the study. N: number

Twenty-nine participants in the DA9301 group and 30 participants in the placebo group completed the final visit evaluation. Treatment compliance was 96.75 ± 5.06 % in the DA9301 group and 95.92 ± 6.60 % in the placebo group (*p* = 0.309, Wilcoxon’s rank sum test). One participant in each group was excluded owing to poor compliance. Two participants in the DA9301 group and five participants in the placebo group were excluded owing to the use of prohibited medicines during the study period. Data from 26 participants in the DA9301 group and 24 participants in the placebo group were used for the final analyses (Fig. 2).

Comparison of demographic characteristics between the two groups showed no significant differences in any parameter except body weight (Table 3).

**Table 3:** Comparison of demographic characteristics between the DA9301 and placebo groups

	Treatment Group				*p*-value
	DA9301		Placebo		
	*n* = 26		*n* = 24		
Sex, n (%)	n	%	n	%	
Male	12	63.16	7	36.84	0.216*
Female	14	45.16	17	54.84	
Smoking, n (%)	n	%	n	%	
no	23 (5)	46.15	23 (2)	53.85	0.312***
yes	3	75.00	1	25.00	
in the past	5	71.43	2	28.57	
Drinking, n (%)	n	%	n	%	
no	8	53.33	7	46.67	0.902*
yes	18	51.43	17	48.57	
Age (yr)					
Mean (SD)	39.85 (11.35)		38.04 (9.92)		0.277**
Median (Min, Max)	38.00 (22.00,64.00)		39.00 (22.00,59.00)		
Height (cm)					
Mean (SD)	166.08 (9.32)		164.02 (9.04)		0.216**
Median (Min, Max)	165.65 (150.10,192.00)		161.30 (150.10,179.40)		
Weight (kg)					
Mean (SD)	66.85 (12.00)		61.05 (12.56)		0.015****
Median (Min, Max)	65.95 (47.70,93.40)		56.25 (45.50,101.30)		

*:*p*-value obtained from Chi-square test. If *p*-value < 0.05, then significant

**:*p*-value obtained from Student’s *t*-test. If *p*-value < 0.05, then significant

***p-value obtained from Fisher’s exact test. If *p*-value < 0.05, then significant

****:*p*-value obtained from Wilcoxon’s rank sum test. If *p*-value < 0.05, then significant

Total asthenopia score (TAS) change induced by tablet computer watching was determined both at the baseline and the final visits. Table 4 presents the changes of TAS by tablet computer watching at the baseline and the final visits. Watching tablet computer at the baseline visit increased TAS by 3.54 and 1.04 in the DA9301 and placebo groups, respectively. The change of TAS in the DA9301 group was statistically significant, while that of placebo group was not significant. It is noteworthy that the mean TAS change at the final visit showed a mean TAS increase of 0.46 and 2.42 in the DA9301 and placebo groups, respectively and this difference was significant (*p* = 0.039, DA9301 baseline visit vs. DA9301 final visit; *p* = 0.020, DA9301 vs. placebo).

**Table 4:** Change in total score of asthenopia questionnaire after tablet computer watching was compared

	Treatment Group		Mean difference [90 % CI]	*p*-value^*^
	DA9301	Placebo		
	*n* = 26	*n* = 24		
Baseline visit				
Mean (SD)	3.54 (6.60)	1.04 (5.30)		
Median (Min, Max)	2.50 (−10.00,24.00)	0.00 (−9.00,17.00)		
Final visit				
Mean (SD)	0.46 (4.50)	2.42 (6.81)		
Median(Min, Max)	1.00 (−12.00,10.00)	0.50 (−8.00,23.00)		
*p*-value**	0.039	0.861		
Mean Change	−3.08	1.38	−4.45 [−8.00,−0.90]	0.020

*:*p*-value obtained from Student,s *t*-test. If *p*-value < 0.05, then significant

**:*p*-value obtained from Paired *t*-test. If *p*-value < 0.05, then significant

We further analyzed each questionnaire of TAS to determine the effect of DA9301. In the DA9301 group, the score changes for 8 of the 10 questions suggested significant improvement and these include questions pertaining to “tired eyes” (*p* = 0.001), “sore/aching eyes” (*p* = 0.038), “irritated eyes’”(*p* = 0.010), “watery eyes” (*p* = 0.005), “dry eyes” (*p* = 0.003), “eye strain” (*p* = 0.006), “blurred vision” (*p* = 0.035), and “visual discomfort” (*p* = 0.018). (Table 5) The placebo group showed improved scores only for “tired eyes” (*p* = 0.002) and “irritated eyes” (*p* = 0.033). It is noteworthy that the score improvement for “dry eyes” showed significant difference when comparing the DA9301 and placebo groups possibly indicating a more prominent DA9301 effect on “‘dry eyes” (*p* = 0.024) (Table 5).

**Table 5:** The change of score for each asthenopia question after tablet computer watching was compared between baseline and final visits

		Treatment Group		*p*-value*
		DA9301	Placebo	
		*n* = 26	*n* = 24	
Tired eyes	Baseline visit			0.124
	Mean (SD)	0.62 (1.10)	0.21 (1.14)	
	Median (Min, Max)	0.00 (−1.00,3.00)	0.00 (−2.00,3.00)	
	Final visit			
	Mean (SD)	−0.31 (1.32)	−0.33 (1.34)	
	Median (Min, Max)	0.00 (−2.00,2.00)	0.00 (−2.00,2.00)	
	*p*-value**	0.001	0.002	
Sore/aching eyes	Baseline visit			0.239
	Mean (SD)	0.23 (0.99)	0.38 (0.92)	
	Median (Min, Max)	0.00 (−3.00,3.00)	0.00 (−1.00,3.00)	
	Final visit			
	Mean (SD)	−0.15 (1.05)	0.21 (1.06)	
	Median (Min, Max)	0.00 (−3.00,1.00)	0.00 (−2.00,2.00)	
	*p*-value**	0.038	0.231	
Irritated eyes	Baseline visit			0.308
	Mean (SD)	0.54 (0.81)	0.54 (1.18)	
	Median (Min, Max)	0.00 (−1.00,2.00)	0.00 (−1.00,3.00)	
	Final visit			
	Mean (SD)	−0.04 (1.22)	0.13 (0.90)	
	Median (Min, Max)	0.00 (−3.00,2.00)	0.00 (−1.00,2.00)	
	*p*-value*	0.010	0.033	
Watery eyes	Baseline visit			0.072
	Mean (SD)	0.81 (1.10)	0.00 (1.59)	
	Median (Min, Max)	0.50 (−1.00,3.00)	0.00 (−4.00,4.00)	
	Final visit			
	Mean (SD)	0.12 (1.07)	−0.21 (1.53)	
	Median (Min, Max)	0.00 (−2.00,4.00)	0.00 (−4.00,3.00)	
	*p*-value*	0.005	0.164	
Dry eyes	Baseline visit			0.024
	Mean (SD)	0.19 (1.39)	0.04 (0.86)	
	Median (Min, Max)	0.00 (−4.00,3.00)	0.00 (−2.00,2.00)	
	Final visit			
	Mean (SD)	−0.69 (1.41)	0.00 (1.18)	
	Median (Min, Max)	−1.00 (−3.00,2.00)	0.00 (−3.00,3.00)	
	*p*-value*	0.003	0.443	
Eye strain	Baseline visit			0.072
	Mean (SD)	0.38 (1.06)	0.08 (1.06)	
	Median (Min, Max)	0.00 (−2.00,3.00)	0.00 (−2.00,2.00)	
	Final visit			
	Mean (SD)	−0.27 (1.08)	−0.13 (1.33)	
	Median (Min, Max)	0.00 (−3.00,2.00)	0.00 (−3.00,3.00)	
	*p*-value*	0.006	0.116	
Hot/burning eyes	Baseline visit			0.201
	Mean (SD)	0.19 (1.27)	0.50 (1.10)	
	Median (Min, Max)	0.00 (−4.00,3.00)	0.00 (−1.00,3.00)	
	Final visit			
	Mean (SD)	−0.15 (1.01)	0.38 (1.21)	
	Median (Min, Max)	0.00 (−3.00,2.00)	0.00 (−2.00,3.00)	
	*p*-value*	0.065	0.189	
Blurred vision	Baseline visit			0.193
	Mean (SD)	0.08 (0.98)	−0.29 (1.04)	
	Median (Min, Max)	0.00 (−3.00,2.00)	0.00 (−3.00,2.00)	
	Final visit			
	Mean (SD)	−0.35 (1.09)	−0.46 (1.35)	
	Median (Min, Max)	0.00 (−3.00,2.00)	0.00 (−3.00,3.00)	
	*p*-value*	0.035	0.191	
Difficult focusing	Baseline visit			0.131
	Mean (SD)	0.46 (0.76)	−0.25 (0.79)	
	Median (Min, Max)	0.00 (0.00,3.00)	0.00 (−3.00,1.00)	
	Final visit			
	Mean (SD)	0.12 (0.82)	−0.25 (0.94)	
	Median (Min, Max)	0.00 (−2.00,2.00)	0.00 (−2.00,3.00)	
	*p*-value*	0.071	0.500	
Visual discomfort	Baseline visit			0.199
	Mean (SD)	0.04 (0.72)	−0.17 (1.05)	
	Median (Min, Max)	0.00 (−1.00,2.00)	0.00 (−3.00,3.00)	
	Final visit			
	Mean (SD)	−0.42 (1.21)	−0.33 (1.66)	
	Median (Min, Max)	0.00 (−3.00,3.00)	0.00 (−5.00,4.00)	
	*p*-value*	0.019	0.279	

*:*p*-value obtained from Student's *t*-test. If *p*-value < 0.05, then significant

**:*p*-value obtained from Paired *t*-test. If *p*-value < 0.05, then significant

There was no significant change of tear film break-up time and total high order wavefront aberration when comparing before and after tablet computer watching in both groups (Tables 6 and 7).

**Table 6:** Change of tear film break-up time (seconds) before and after tablet computer watching was compared

	Treatment Group					
	DA9301			Placebo		
	*n* = 26			*n* = 24		
	Before video stimulus	After video stimulus	*p*-value	Before video stimulus	After video stimulus	*p*-value
Baseline visit						
Mean (SD)	4.90 (1.50)	4.60 (1.21)	0.893	5.20 (1.55)	4.62 (1.46)	0.989
Median (Min, Max)	4.50 (3, 8)	4.30 (3, 7.3)		5.15 (3, 9.3)	4.15 (2.3,9.3)	
Final visit						
Mean (SD)	4.78 (1.50)	4.34 (1.04)	0.963	4.67 (1.20)	4.35 (0.92)	0.972
Median (Min, Max)	4.65 (2.3,7.7)	4.15 (3,7)		4.5 (2.7,7.3)	4.3 (3,6.7)	

*p*-value obtained from one-sided paired *t*-test. If *p*-value < 0.05, then significant

**Table 7:** Change of total high order wavefront aberration (μm) before and after tablet computer watching was compared

	Treatment Group					
	DA9301			Placebo		
	*n* = 26			*n* = 24		
	Before video stimulus	After video stimulus	*p*-value	Before video stimulus	After video stimulus	*p*-value
Baseline (Visit 2)						
Mean (SD)	2.33 (2.05)	2.32 (1.90)	0.451	2.51 (2.46)	2.61 (2.62)	0.856
Median (Min, Max)	1.55 (0.31,7.08)	1.7 (0.23,6.50)		1.49 (0.36,9.14)	1.4 (0.35,9.97)	
Visit 3						
Mean (SD)	2.18 (1.83)	2.27 (1.93)	0.715	2.49 (2.36)	2.53 (2.33)	0.633
Median (Min, Max)	1.53 (0.42,5.95)	1.7 (0.27,6.92)		1.92 (0.27,7.69)	1.8 (0.36,7.74)	

*p*-value obtained from one-sided paired *t*-test. If *p*-value < 0.05, then significant

There were no significant ocular and systemic complications related to this study in both DA9301 and control groups. Dietary antioxidant consumption between two groups showed no significant difference (Table 8).

**Table 8:** Comparison of dietary antioxidant components between DA9301 and placebo group during the last three days before the 4-week visit

	DA9301 (*n* = 26)	Placebo (*n* = 24)	*p*-value
Vitamin C (mg)			
Mean (SD)	177.94 (85.87)	209.02 (114.14)	0.275
Median (Min, Max)	171.81 (54.72, 369.20)	188.58 (58.98, 577.18)	
Vitamin E (mg)			
Mean (SD)	37.01 (12.50)	36.72 (12.62)	0.935
Median (Min, Max)	35.11 (19.16,62.04)	33.53 (18.28,76.56)	
ß- carotene (mg)			
Mean (SD)	7771.03 (4323.44)	7342.58 (2939.67)	0.682
Median (Min, Max)	6976.01 (1407.43, 18856.67)	6775.62 (2432.26,14648.82)	
Selenium (μg)			
Mean (SD)	239.75 (77.62)	214.80 (55.19)	0.193
Median (Min, Max)	234.90 (126.29,383.19)	214.60 (128.88,361.70)	

*p*-value obtained from two sample *t*-test. If *p*-value < 0.05, then significant

## Discussion

Recent advances in smart mobile devices have increased the popularity of smart phones and tablet computers among the general population. With the wide-spread use of smart mobile devices, smart device addiction has become serious health problem worldwide [6, 26]. Continuous accommodation is necessary to focus on the display of smart mobile devices for an extended period. The fatigue induced by video monitors, such as LCD and LED monitors, has been previously referred as computer vision syndrome (CVS) [4, 5, 27, 28]. About 64–90 % of computer users develop CVS [4]. It has been reported that blink rate during computer work significantly decreases and negatively correlates with eye discomfort score [10]. Another study revealed that computer work for more than 4 h significantly increased eye discomfort [29]. In addition, electronic book readers with LCD monitor cause marked visual fatigue [30].

Our results have demonstrated the aggravation of CVS upon viewing monitors such as those of mobile devices. A recent study demonstrated that the preferred distance for viewing a mobile device (36.2 cm) is shorter than the typical distance for reading books (40 cm) and this requires more accommodation and convergence [31]. The cumulative effect of continuous accommodation and convergence can manifest as various ocular, visual and musculoskeletal symptoms which are collectively called as asthenopia.

Various methods of treatment have been suggested to manage CVS. These include correction of residual refractive errors, instillation of artificial tears, combination of specific body posture, breathing and mental control, visual ergonomics and Ayurveda medicine [28, 32–34]. There are anecdotal reports about the effectiveness of these treatment modalities. Specific pharmacologic intervention to alleviate asthenopia induced by CVS has been investigated previously. Omega-3 fatty acid was reported to decrease visual discomfort in CVS by decreasing tear evaporation and improving dry eye symptoms [35]. Topical brief application of polyphenol on eyes alleviated eye fatigue after computer work [36]. It is also important to maintain baseline efforts such as taking regular break, proper refractive correction, and treating underlying ocular surface disease to prevent CVS.

In this study, we found that oral intake of DA9301 for 4 weeks significantly alleviated visual discomfort induced by tablet computer work. The exact mechanism of the action of DA9301 needs further investigation. However, as mentioned earlier, DA9301 contains antioxidant ingredients such as anthocyanins and flavonoids [31]. It has been recently reported that *Vaccinium uliginosum* extract prevented the blue light-induced human retinal pigment epithelial cell death by inhibiting photo-oxidation of N-retinyl-N-retinylidene ethanolamine [37]. Extract of *V. uliginosum* partially preserved the outer nuclear layer of retina after light damage [19]. Another study reported the positive effect of *V. uliginosum* extract on improving learning and memory impairment in murine Alzheimer’s disease model [38]. The symptoms of dry eye disease and asthenopia partially overlap [3]. Previous studies have demonstrated the beneficial effect of antioxidants in alleviating dry eye disease [16–18]. We hypothesize that a similar mechanism might be involved in the DA9301-induced alleviation of asthenopia in our study.

There are several limitations of this study. First, we used questionnaires to evaluate the effect of DA9301. The responses to the questionnaire are somewhat subjective and could be affected by responders’ daily physical and mental condition. It would be better to adopt an objective measurement of asthenopia. It is noteworthy that a new questionnaire to assess computer vision symptom was recently developed and now is under validation [39]. However, to the best of our knowledge, there is no established objective method to quantify asthenopia, although tear film break-up time and wavefront aberration were used as an possible objective method in our study. Second, the tablet computer stimulus employed in our study is shorter (1 h) than that in a typical CVS model (several hours of intensive computer work). This could explain the relatively mild TAS increase observed in our study. More prolonged and intense computer work could aggravate TAS. Another limitation of this study is the lack of eye exercises screening of the participants. Recently, awareness regarding the eye disorders is on the rise and many people are doing regular eye exercises which can have significant impact on the study. In addition, although DA9301 was found to significantly alleviate asthenopia induced by table computer watching, a reverse trial of DA9301 and placebo (applying DA9301 in placebo group and vice versa) after a washout period may significantly enhance the strength of our result.

## Conclusions

Oral intake of DA9301 was effective in alleviating CVS induced by a tablet computer and this could be attributed to its strong antioxidant potential. Further investigation of the mechanisms underlying the effect of DA9301 is warranted.
